# The Importance of Quantitative Trace Analysis in Industry Today

**DOI:** 10.6028/jres.093.015

**Published:** 1988-06-01

**Authors:** Bernard J. Bulkin

**Affiliations:** Director, Analytical Sciences Laboratory, BP America Research & Development, Cleveland, OH 44128

Trace analysis in the industrial analytical laboratory represents a substantial portion of the overall lab workload. This paper will explore some of the kinds of analyses that are done, why they are done, and what the limitations are to our current capabilities.

“Industry” represents the full diversity of analytical problems, and even within one company, such as BP America, important trace quantitation problems can arise from petroleum products, ceramics, minerals, biological materials, chemicals, catalysts, and, of course, environmental samples relating to all of these. It would be impossible to review all of the trace analytical methods used for such a diverse set of samples, to compare relative merits, or to in any way survey the field of trace analysis in industry. Rather, this paper looks at certain major themes that are driving the entire area, overlapping many techniques in each case.

For all of the diversity of quantitative industrial trace analysis, three motivational themes seem to dominate: samples where the trace quantity provides the value to a product; samples where trace species degrade the value of a product; and, samples where trace species represent a potential hazard to human, animal or plant life. In suggesting these categories, and in the subsequent discussion, I am removing from the realm of trace analysis problems in which the analyte is in high concentration but small quantity. For some techniques these may present the same problem as low concentration trace analysis, but increasingly, we find that instrumentation has evolved considerably in the ability to handle small samples.

The first category, trace quantities as key to product value, is probably most noticeable in importance today for the electronics industry. While classical chemistry recognized the importance of certain trace elements or compounds, it was the electronics industry, beginning in the earliest days of the transistor and continuing to the present, that turned this into a picoscience.

Examples in other aspects of chemistry and materials science abound. Modern heterogeneous catalysts have derived selectivity and activity from trace elements. Metallurgists, ceramists, and polymer scientists can bring about significant improvements in material properties by adding trace quantities of appropriate substances.

The analytical challenge here has been considerable. In many cases we are asked to analyze very low levels, with a high degree of quantitation, and on solid phases. It is the latter point that probably has provided the greatest challenges and the most innovative solutions. The triumphs of SIMS, an industrial innovation, despite its being a destructive technique, are to be particularly noted in this field. Many advances have also come from Fourier transform techniques, in NMR, IR, and mass spectrometry. Even in such well developed areas as atomic spectroscopy, the introduction of low temperature plasma ashing and acid digestion using sealed Teflon vessels has permitted dissolution of solids with almost no loss or contamination.

There still remains much to be done. While SIMS provides some depth profiling, we continue to be challenged by deep interfaces. These are of great importance, but analytical methodology for reaching and analyzing them is decades behind our ability to deal with surfaces.

The second area is also a well established problem in the electronics industry, but pervades much of the rest of chemistry. While some trace elements provide catalyst selectivity, others are potent catalyst poisons. Relatively low levels of metals in coke (iron, nickel, vanadium) result in substantial drops in selling price. We see cases of all sorts where it is necessary to find *analytical* methods that will aid in anticipating the trace levels that will degrade quality of a product.

In many respects, the analytical problems associated with this category are not distinct from those of the first group of samples. Once again, for example, we see the value of analytical techniques such as SIMS. Other techniques, well established years ago, are experiencing varying degrees of revival to meet the analytical challenges of difficult samples. Neutron activation analysis, particularly for refractory materials, is being increasingly used. In the electronics industry, the degree of natural radioactivity in materials is an important property, and analytical chemists are being called upon increasingly to revive what may be long dormant knowledge of radiochemical methods.

Laser analytical methods are also becoming far more important. There are, for example, electrodeposition processes that will concentrate the trace element in a plating bath from the ppb level to the ppm level in the final device. It is necessary to find methods that will analyze the bath, and even monitor the depletion kinetics of the trace elements. Laser methods often are suitable here.

And we should not forget tried and true electrochemical or wet methods. As will be discussed in more detail below, such methods are particularly amenable to being carried out with laboratory robots, leading to high quality results and low cost for repetitive analysis.

The third category probably represents a large, distinct sub-discipline of analyses—environmental trace analysis, that is best dealt with separately. There is, to be sure, a set of established methodologies here. Most are considered to be adequate in terms of sensitivity, although there is certainly considerable merit to many of the active research projects in developing new methodologies for trace analysis of environmentally important species.

What is a problem, and a major one, in environmental trace analysis, is the analytical reproducibility of the established methods. Even more important than standard deviations within a single laboratory running replicate samples is the interlaboratory variance. Several publications, mostly citing older studies, have already documented the extent of this problem. The problem is also made evident by looking at the standards for interlaboratory comparison of results in the actual regulations. The extent of the problem is confirmed by recent interlaboratory tests conducted by government or independent industry groups, and by our own evaluation of contract analytical laboratories.

One approach to attacking this problem is increased use of laboratory robotics in environmental trace analysis. The original motivation for the application of laboratory robots to this area was to reduce costs and sample turnaround time. These motivations are valid, and straightforward economic analysis demonstrates that laboratory robots have a rapid payback in environmental trace analysis.

However, the impact of the laboratory robot on improved quantitation may, in the end, be as important a reason for adopting this approach. Again, there are two aspects. First, the robot carries out all operations in a far more reproducible manner than can several different human analysts, and thus directly leads to better analytical results. This will be demonstrated with the specific example of purge-and-trap analysis of volatile organics. Second, even when the robot is not to be used for an analysis on a long term basis, it is a powerful tool for method development. Using a laboratory robot, it is possible to explore the parameter space of an analytical method rather quickly and systematically, to minimize imprecision and eliminate sources of variation/bias. The optimized method, even if ultimately performed by humans, then gives improved results.

Robots are also very amenable to improved quality control in environmental analysis. Automated blanks and spiking stations have been designed and applied to these analyses. But one can go beyond this, to eliminate the biases that appear to creep into QC, by having the robot carry out blanks, spiked samples, etc., on a random basis. This should greatly improve data quality.

We recognize that in most large companies, our field of endeavor is now best described as Analytical Sciences. There is a major role for chemistry, but also roles for materials science, physics, engineering, and statistics. We need a diverse set of skills, and an ongoing research effort to keep up with the problems generated by industrial products. It seems likely that in pursuing this research effort, trace analysis will be an ongoing theme for a long time to come.

